# EyeInvaS: Lowering Barriers to Public Participation in Invasive Alien Species Monitoring Through Deep Learning

**DOI:** 10.3390/ani15213181

**Published:** 2025-10-31

**Authors:** Hao Chen, Jiaogen Zhou, Wenbiao Wu, Changhui Xu, Yanzhu Ji

**Affiliations:** 1 School of Geography and Planning, Huaiyin Normal University, Huai’an 223300, China; chenhao@hytc.edu.cn; 2 Research Center of Information Technology, Beijing Academy of Agriculture and Forestry Sciences, Beijing 100097, China; wuwb@nercita.org.cn; 3 Chinese Academy of Surveying & Mapping, Beijing 100036, China; chxu@casm.ac.cn; 4 State Key Laboratory of Animal Biodiversity Conservation and Integrated Pest Management, Institute of Zoology, Chinese Academy of Sciences, Beijing 100101, China; jiyanzhu@ioz.ac.cn

**Keywords:** invasive alien species, deep neural networks, citizen science, ecological monitoring, image recognition, mobile application, species recognition system

## Abstract

Invasive alien species (IASs) pose escalating threats to global ecosystems, biodiversity, and human well-being. Public participation in IAS monitoring is often limited by taxonomic expertise gaps. To address this, we established a multi-taxa image dataset covering 54 key IAS in China, benchmarked nine deep learning models, and quantified impacts of varying scenarios and target scales. EfficientNetV2 achieved superior accuracy, with F1-scores of 83.66% (original dataset) and 93.32% (hybrid dataset). Recognition accuracy peaked when targets occupied 60% of the frame against simple backgrounds. Leveraging these findings, we developed EyeInvaS, an AI-powered system integrating image acquisition, recognition, geotagging, and data sharing to democratize IAS surveillance. Crucially, in a large-scale public deployment in Huai’an, China, 1683 user submissions via EyeInvaS enabled mapping of *Solidago canadensis*, revealing strong associations with riverbanks and roads. Our results validate the feasibility of deep learning in empowering citizens in IAS surveillance and biodiversity governance.

## Simple Summary

Invasive species pose serious threats to global biodiversity, agriculture, and ecosystems. Public participation offers an effective way to achieve large-scale and long-term monitoring, yet limited professional knowledge often reduces identification accuracy. This study introduces EyeInvaS, an intelligent image recognition system that enables citizens to identify and monitor invasive species simply by taking photos with their mobile phones. Using over ten thousand images—collected from online sources and synthetically generated under different scales and backgrounds—we built nine representative recognition models based on transfer learning and identified the optimal model and target scale through comparative analysis. The integrated EyeInvaS system supports key functions such as field reporting, rapid recognition, geographic tagging, and data sharing. Its reliability was validated through real-world field investigations of *Solidago canadensis* in Huai’an, China. This study demonstrates how deep learning technology can empower public participation in ecological protection and improve the efficiency of early detection and monitoring of invasive species.

## 1. Introduction

The accelerating pace of globalization and increased cross-border transportation have significantly facilitated the unnatural spread of species across geographic boundaries [1]. In the absence of natural enemies or ecological constraints, alien species with high adaptability and reproductive capacity can readily establish invasive populations, thereby disrupting local ecosystems over the long term [2]. According to recent estimates, more than 37,000 invasive alien species have been identified globally, spanning a wide taxonomic range from vascular plants and vertebrates to insects, mollusks, and microorganisms, reflecting an increasingly multi-taxa and multi-niche invasion pattern [3].

The ecological, economic, and health-related risks posed by IAS have emerged as a global policy concern [4]. These species often outcompete natives for resources and niches, leading to ecosystem degradation and the collapse of native communities [5,6]. Economically, IAS result in billions of dollars in losses annually across agriculture, forestry, fisheries, and aquaculture sectors [7,8]. Certain species also act as carriers of novel pathogens, posing increased risks to public health [9]. Given these threats, early detection and rapid species identification are essential for reducing response costs and enabling targeted interventions.

Although traditional monitoring technologies are scientifically rigorous, they face critical limitations when applied at large spatial scales or in time-sensitive contexts. Environmental DNA (eDNA) methods are constrained by primer design and degradation rates [10,11]; remote sensing lacks the resolution needed for ground-level species detection [12]; and chemical baiting approaches are susceptible to background noise and temporal variability [13]. In response, public participation has become a valuable means to broaden the scope of invasive species monitoring. However, limited taxonomic knowledge among participants often restricts the effectiveness of such efforts, making it difficult to achieve reliable species identification [14]. Global citizen science platforms like iNaturalist [15] and EDDMaps [16], while advancing crowdsourced IAS monitoring, fail to meet China’s needs by lacking sufficient coverage of the country’s regional IAS and facing hours-long review delays from expert verification, which slows the critical early response to IAS.

Recent advancements in deep learning and computer vision offer new opportunities to enhance species identification by non-expert users. From convolutional neural networks (CNNs) to attention-based Transformers, these models have advanced in their ability to extract complex semantic information from images [17,18,19,20]. Lightweight architectures such as MobileNet and EfficientNet further enable deployment on mobile and edge devices, supporting real-time inference in field conditions [21,22,23]. These models have already shown efficacy in tasks such as species classification [24], ecological monitoring [25,26], and plant disease diagnosis [27,28].

Nevertheless, applying AI-based recognition to citizen science monitoring of IAS faces two major challenges: first, existing tools are often not user-friendly or accessible to the general public; second, field images frequently involve complex backgrounds and variable target scales, which degrade model robustness and accuracy.

To address these challenges, we propose EyeInvaS, a deep learning-powered intelligent recognition system designed to enhance public involvement in ecological surveillance. Our methodology focused on (1) establishing an image database covering high-concern invasive species in China; (2) comparative evaluation of nine recognition models; and (3) quantitative assessment of scale and background interference effects. The resulting framework synergizes public-submitted imagery with image acquisition, species recognition, geotagging, and data sharing functionalities, effectively bridging the gap between public participation and intelligent ecological monitoring. We validated this approach in a real-word *Solidago canadensis* surveillance initiative. This work provides a scalable and replicable tool for global IAS surveillance while advancing the integration of citizen participation into biodiversity governance.

## 2. Data and Methods

### 2.1. Dataset Construction and Preprocessing

#### 2.1.1. Original Dataset

Based on the January 2023 edition of the List of Key Managed Invasive Alien Species in China, we selected 54 species across six major taxonomic groups—plants, insects, mollusks, fishes, amphibians, and reptiles—that are feasible for image acquisition. Microorganisms were excluded due to their invisibility to the naked eye and limited relevance for citizen detection tasks.

We developed a Python-based web crawler (version 3.12.3) to collect image samples of the target species from Baidu Images and Google Images, using their common names and scientific names. Low-quality and misidentified images were removed through manual screening, and taxonomy experts conducted a secondary review to ensure accuracy. The final dataset included 6109 images, each annotated with metadata including taxonomy, ecological traits, and geographic distribution. These data were also used to populate species profiles in the mobile application (see Table A1).

#### 2.1.2. Multi-Scale and Multi-Background Synthetic Dataset

To assess model robustness under varying environmental conditions, we constructed a synthetic dataset simulating different scenarios and target scales. Specifically, we curated a library of scenario images representing 9 typical habitat types (e.g., riverbanks, forests, hillsides, farmland, and grasslands), standardized to a resolution of 224 × 224 pixels. For each of the 54 species, we prepared silhouette images of the target organism and resized them into 9 scale levels (from 25 × 25 to 200 × 200 pixels), simulating different observation distances.

**Figure 1:**
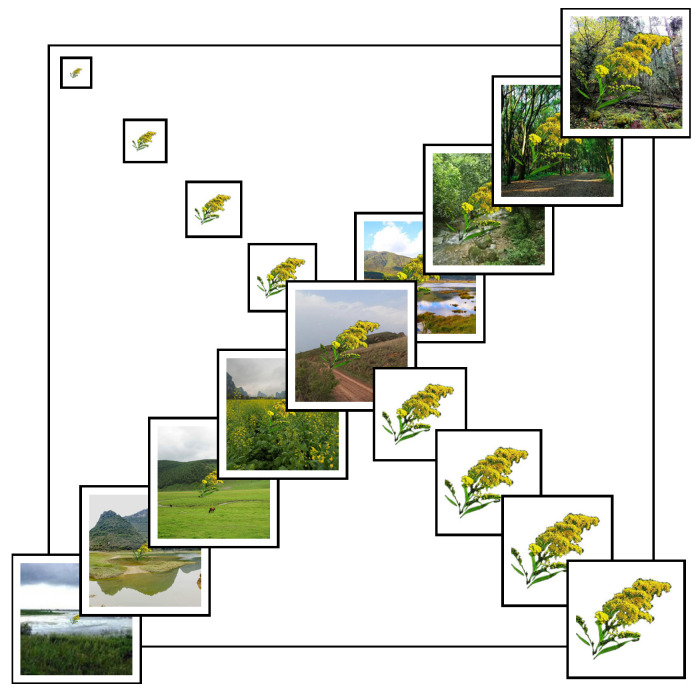
Multi-scale and multi-background synthetic images of *Solidago canadensis*. The main diagonal displays 9 ecological scenarios (each 224 × 224 pixels), while the sub-diagonals show 9 target object scales ranging from 25 × 25 to 200 × 200 pixels. This matrix was used to simulate variation in field observation conditions.

Using image composition techniques, each target image was overlaid onto 9 background images without transparency to simulate real-world complexity. In total, 4374 synthetic samples were generated, each representing a unique combination of background and target scale. Figure 1 illustrates the synthetic image set for *Solidago canadensis*.

#### 2.1.3. Data Augmentation

To improve model generalization and reduce overfitting, we applied geometric data augmentation techniques to the images. These included random rotations and horizontal and vertical flips, thereby increasing sample diversity and enhancing model robustness.

### 2.2. Model Development and Performance Evaluation

#### 2.2.1. Model Selection and Training Strategy

We selected 9 representative deep learning architectures covering both convolutional and transformer-based paradigms, ranging from classic high-capacity models to efficient mobile-friendly networks. These include the early CNN AlexNet and deeper models such as VGG16 [29], ResNet50 [18], and DenseNet161 [30], which differ in depth, connection strategies, and feature reuse mechanisms. For mobile deployment, we evaluated lightweight architectures including MobileNetV2 [23], ShuffleNetV2 [21], and EfficientNetV2 [22]—designed for high efficiency with minimal performance trade-offs. Finally, we incorporated two transformer-based models, Vision Transformer (ViT) [19] and Swin Transformer (SwinT) [20], which model global and hierarchical attention mechanisms, respectively. This selection allows us to comprehensively benchmark performance across varying network designs and computational demands.

All models were initialized using ImageNet pre-trained weights and fine-tuned via transfer learning. Specifically, the feature extraction layers were frozen, and only the classification layers were retrained to adapt to the multi-class IAS recognition task. The dataset was split into training, validation, and testing subsets at a ratio of 8:1:1. Training was conducted over 100 epochs using the Adam optimizer with an initial learning rate of $1\times 10^{-4}$, a weight decay of $1\times 10^{-5}$ and a batch size of 32, with categorical cross-entropy as the loss function. All experiments were performed on a device with an NVIDIA RTX 4090 GPU.

#### 2.2.2. Evaluation Metrics

Model performance was comprehensively evaluated using four standard metrics: Accuracy, Precision, Recall, and F1-Score, defined as follows:

(1) $$Accuracy=\frac{TP+TN}{TP+TN+FP+FN},$$

(2) $$Precision=\frac{TP}{TP+FP},$$

(3) $$Recall=\frac{TP}{TP+FN},$$

(4) $$F1\mathrm{-s}core=2\times \frac{precision\times recall}{precision+recall}.$$

Here, TP, TN, FP, and FN represent true positives, true negatives, false positives, and false negatives, respectively. Among these metrics, the F1-score—balancing precision and recall—was used as the primary indicator for model comparison in this multi-class classification task.

### 2.3. EyeInvaS: An Intelligent Recognition System for IAS

**Figure 2:**
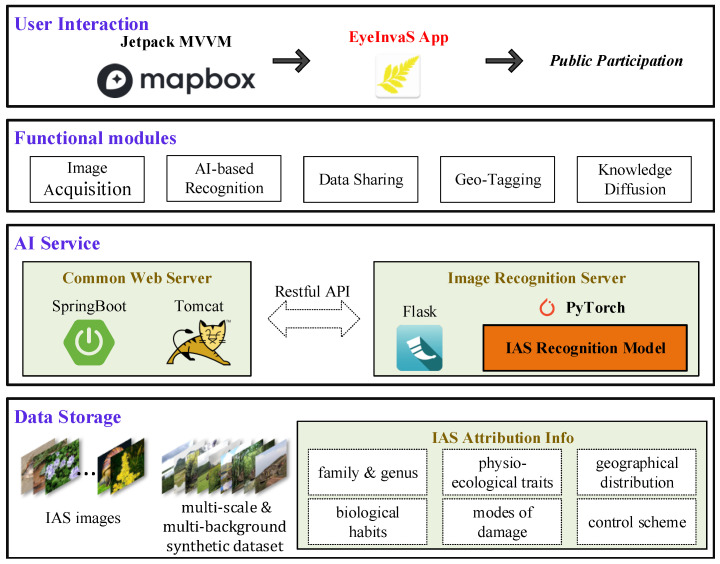
Four-tier architecture of the EyeInvaS platform: data storage, AI services, functional modules, and user interaction layers.

To facilitate real-world deployment and enhance public participation in monitoring, we developed a mobile application system named EyeInvaS. The overall system architecture is shown in Figure 2 and comprises the following four layers:

1. Data storage: Responsible for storing structured information, including invasive species images, taxonomic labels, and biological trait metadata.
2. AI Service: Composed of general web services (based on SpringBoot) and the AI model service (built with PyTorch (2.5.1) and Flask (3.1.0)). These components communicate through RESTful APIs, ensuring modularity and extensibility.
3. Functional modules: Integrates key functional modules such as image acquisition, IAS recognition, data sharing, location tagging, and knowledge diffusion. These modules were designed based on a user survey identifying public priorities in invasive species detection.
4. User interaction: Represented by the EyeInvaS mobile application, which supports visual recognition of invasive species and serves as the main user interface. The app is built using the Jetpack MVVM architecture to improve code maintainability and device compatibility. It incorporates Mapbox for geolocation and spatial visualization.

Users can take photos or upload images from their gallery. The app then crops the image to an appropriate size (see Section 3.1.3), and calls the AI recognition service model to return a recognition result.

## 3. Results and Applications

### 3.1. Results

#### 3.1.1. Model Performance Comparison

**Figure 3:**
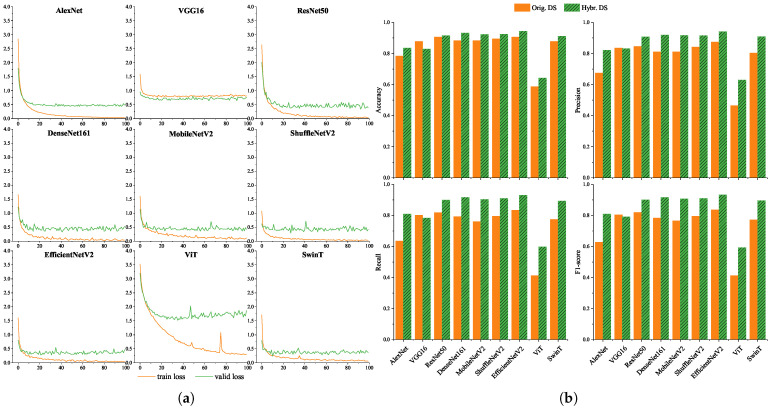
Training loss dynamics and classification performance of 9 deep learning models. (**a**) Train loss and valid loss. Except for ViT, the loss curves of all other models show a stable convergence trend. (**b**) Classification performance on original and hybrid datasets. Comparative performance on original and hybrid datasets: Accuracy, Precision, Recall, and F1-score. EfficientNetV2 demonstrates superior performance across both datasets, with F1-scores of 83.66% on the original dataset and 93.32% on the hybrid dataset.

To evaluate model robustness under varying image conditions, we trained and evaluated nine models on both the original dataset and the hybrid dataset (original + synthetic). The training loss and validation loss curves of the 9 models on the hybrid dataset are shown in Figure 3a. Under the 100-epoch training strategy, the training loss of all models showed a downward trend, which indicates all models could effectively fit the training data. Except for VGG16 and ViT, the training loss of other models finally converged to below 0.1, and the gap between their training loss and validation loss was always less than 0.5, with no obvious overfitting. The train loss of VGG16 also decreased, but its final convergence value was higher than 0.1, resulting in a relatively weak convergence effect. The loss of ViT fluctuated significantly during the downward process. In contrast, SwinT, which is also a Transformer model, had a smoother loss curve and better performance.

Figure 3b presents the performance of each model in terms of accuracy, precision, recall, and F1-score. Overall, all models performed better on the hybrid dataset than on the original dataset, suggesting that synthetic augmentation effectively enhanced model generalization. CNN-based models demonstrated improved performance with increasing network depth. Lightweight networks such as MobileNetV2, ShuffleNetV2, and EfficientNetV2 achieved a good balance between computational efficiency and classification accuracy. Among the Transformer-based models, SwinT performed comparably to mainstream CNNs, while ViT showed suboptimal results—likely due to its reliance on large training datasets [20]. EfficientNetV2 achieved the highest F1-scores (83.66% and 93.32%) on both datasets and was chosen as the system backbone for its strong performance and mobile deployability.

Considering real-world monitoring scenarios, the public may encounter IAS from different taxonomic groups such as plants, insects, and amphibians. The overall excellent performance of the model on a single dataset does not fully indicate consistent recognition stability across various taxonomic groups—for instance, taxonomic groups with significantly distinct morphological features (e.g., amphibians and plants) may impose different requirements on the model’s feature extraction capability. To further verify the applicability of EfficientNetV2 in diverse taxa and clarify its performance differences and potential limitations among different taxa, we conducted an in-depth analysis of its cross-taxonomic recognition performance on the test dataset (see Table 1).

From the perspective of cross-taxonomic performance results, EfficientNetV2 also demonstrated strong classification capability. Amphibians, as the only taxonomic group containing a single species (*Lithobates catesbeianus*), had highly distinguishable morphological features (such as tympanic membranes and webbed feet) across different growth stages and shooting angles, with extremely high annotation consistency. Consequently, its Accuracy, Precision, Recall, and F1-score all reached 1.00. The F1-scores of fishes and reptiles were also outstanding, both standing at 0.98. Even though there were very few misclassifications, their unique morphological features (such as body shape and scale structure) still ensured near-perfect recognition performance. The F1-score of mollusks was 0.97, showing stable overall performance. The Accuracy of plants and insects was both 0.93, slightly lower than those of other groups. This difference mainly stems from the complexity of species within these two groups—the plant group includes 33 species (16 of which belong to the Asteraceae family), and the insect group includes 13 species. The morphological similarity among closely related species (e.g., the leaf morphology and inflorescence structure of *Bidens pilosa* and *Chromolaena odorata*) easily leads to intra-group misclassifications by the model.

#### 3.1.2. Model Explainability and Prediction Visualization

**Figure 4:**
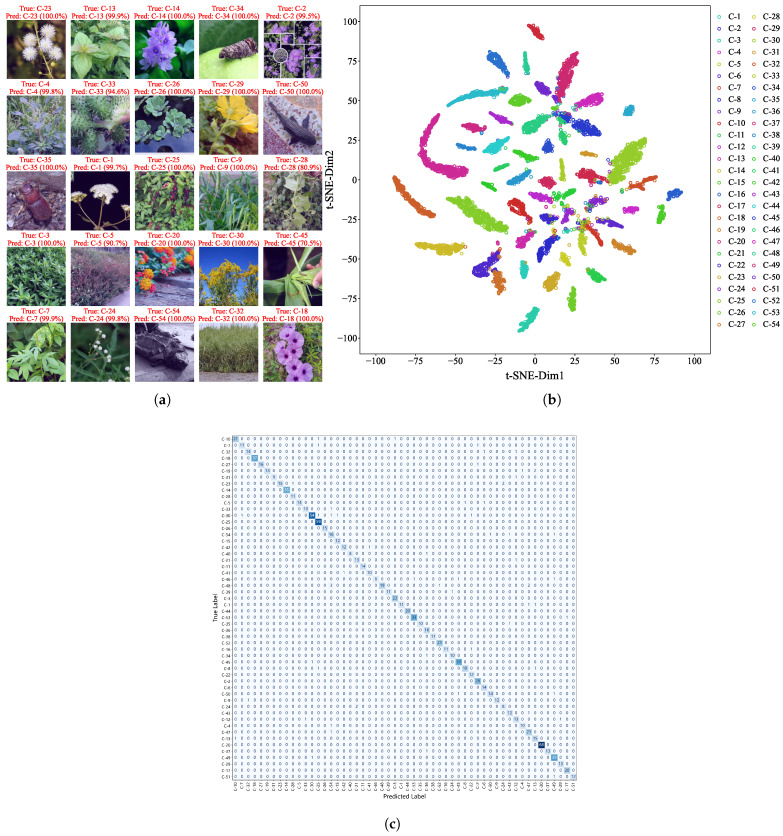
Visualization of recognition results. (**a**) High-confidence predictions on test images with annotated true labels, predicted labels, and probabilities. (**b**) t-SNE Visualization of EfficientNetV2 features from the hybrid dataset. Distinct clustering patterns reflect species discriminability, validating model reliability for invasive species identification. (**c**) Confusion matrix of EfficientNetV2 for 54 IAS: the vertical axis represents true labels, the horizontal axis represents predicted labels, and the diagonal represents correct classifications.

Among the 1048 images in the test dataset from the hybrid dataset, EfficientNetV2 achieved an overall prediction accuracy of 94.36%, with only 60 misclassifications. Figure 4a shows examples of correctly identified species with high confidence scores, often approaching 100%. To further examine the model’s internal representations, we visualized the feature distribution using t-distributed stochastic neighbor embedding (t-SNE) on the hybrid dataset (Figure 4b). Features from the same species formed tight clusters, while those from different species were clearly separable, confirming the model’s strong class discriminability.

We also employed Grad-CAM and Guided Grad-CAM to visualize the attention regions in misclassified samples (see Table A2). Results indicated that errors were primarily caused by viewing angle bias, small target scale, or background interference. For instance, in the case of *Ipomoea cairica*, the model focused on the inflorescence while ignoring leaf characteristics, resulting in a misclassification as *Phytolacca acinosa*.

To systematically reveal the confusion relationships among all 54 IAS, we constructed a species-level confusion matrix (Figure 4c). This matrix presents the number of correct and incorrect predictions for each species, with the vertical axis representing true labels and the horizontal axis representing predicted labels. It can be clearly seen that misclassifications are mainly concentrated among congeneric or confamilial species—for example, *Ambrosia trifida* and *Solidago canadensis*, both belonging to Asteraceae; *Cydia pomonella* and *Hyphantria cunea*, both with similar characteristics of Lepidopteran insects—which is consistent with the conclusion of “misclassifications caused by morphological similarity” in the Grad-CAM analysis.

These insights informed the design of user guidelines: we recommend capturing complete, well-lit images that highlight key morphological features (e.g., leaves, stems, flower structure) and avoiding close-ups or backlit shots that may obscure relevant details.

#### 3.1.3. Effects of Target Scale and Background Complexity

**Figure 5:**
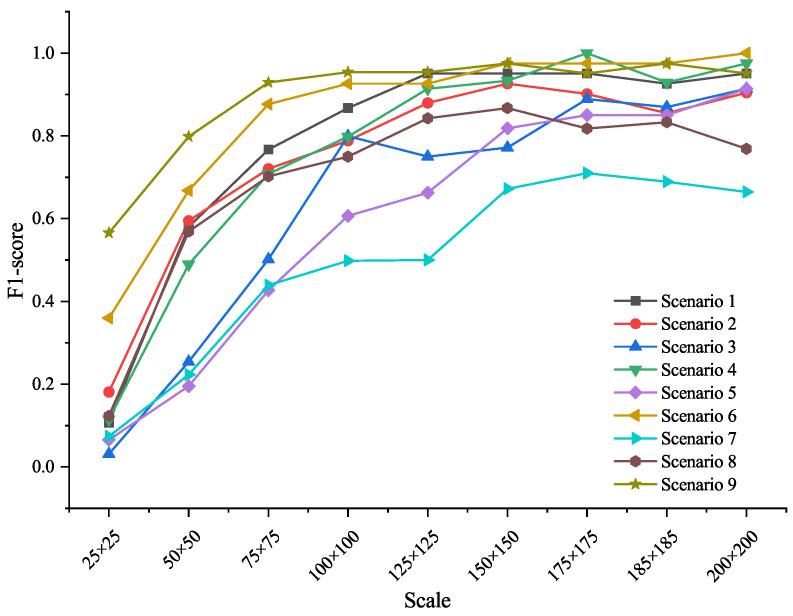
F1-Score of EfficientNetV2 across background and target scales. Performance improved with increasing target size, while complex backgrounds reduced accuracy due to texture interference.

To evaluate the influence of target size and environmental background, we tested model performance of EfficientNetV2 across the 9 synthetic scales and background types. For each species, synthetic images were generated under nine different sizes and nine background scenarios. Figure 5 presents F1-score variations across these conditions.

Results show that target scale significantly affected recognition accuracy. When the object size was below 100 × 100 pixels, model performance dropped sharply. Accuracy stabilized and peaked when the target covered approximately 61% of the image area (around 175 × 175 pixels). Background complexity also played a role: recognition tended to be lower in cluttered environments (e.g., forest floors, agricultural fields), likely due to visual distraction from irrelevant textures.

These findings were incorporated into the EyeInvaS app by implementing a framing guide that encourages users to capture images in which the target occupies at least 60% of the frame and by avoiding complex or noisy backgrounds to improve recognition accuracy.

### 3.2. Application Scenarios

#### 3.2.1. Functional Modules of the EyeInvaS App

**Figure A1:**
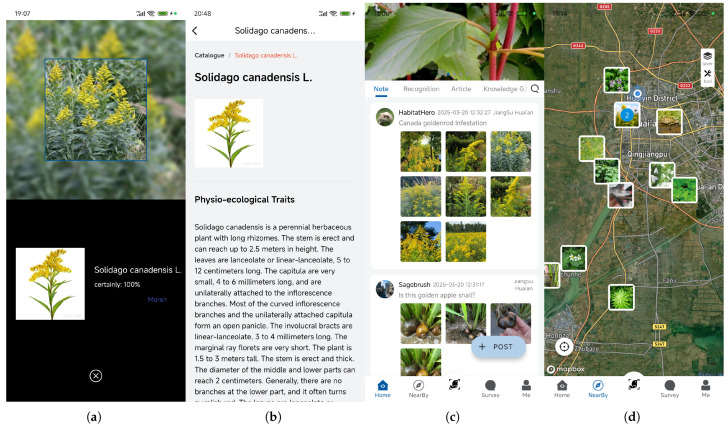
Core functional modules of the EyeInvaS app. (**a**) Image recognition; (**b**) Species information; (**c**) Data sharing; (**d**) Geo-Tagging. See Supplementary Video S1 for a live demonstration of the app interface and workflow.

Figure A1 presents the following main functional modules of the EyeInvaS app designed for citizen engagement:

1. AI-based Image Recognition: Users can take photos or upload existing images for recognition. A built-in framing guide helps users compose images that meet the model’s optimal input conditions. The system returns the predicted species name and confidence score.
2. Species Information: Users can access detailed information about the identified species, including taxonomy, ecological impact, geographic distribution, and recommended management strategies, enhancing public knowledge and awareness.
3. Data Sharing: Users may add time and location metadata to their observations and upload them to the database, enabling both personal record-keeping and crowdsourced data aggregation.
4. Geotagging: Integrated with Mapbox, this feature visualizes uploaded observations as geospatial points, making spatial patterns and invasion hotspots easily interpretable.

These features together form a closed-loop workflow from image acquisition to spatial visualization, empowering the public to participate meaningfully in IAS monitoring. A short demonstration video of the EyeInvaS app, highlighting core functions such as image capture, species recognition, data sharing, and spatial visualization, is available as Supplementary Video S1.

#### 3.2.2. Case Study: Monitoring *Solidago canadensis* in Huai’an, China

To evaluate real-world usability, we conducted a pilot deployment of the EyeInvaS app in Huai’an, China. We collaborated with the local “SmartEye” environmental protection group to recruit volunteers. Participants were briefed on the use of the EyeInvaS app and instructed to document occurrences of *Solidago canadensis* by photographing plants and uploading records with location data.

**Figure 6:**
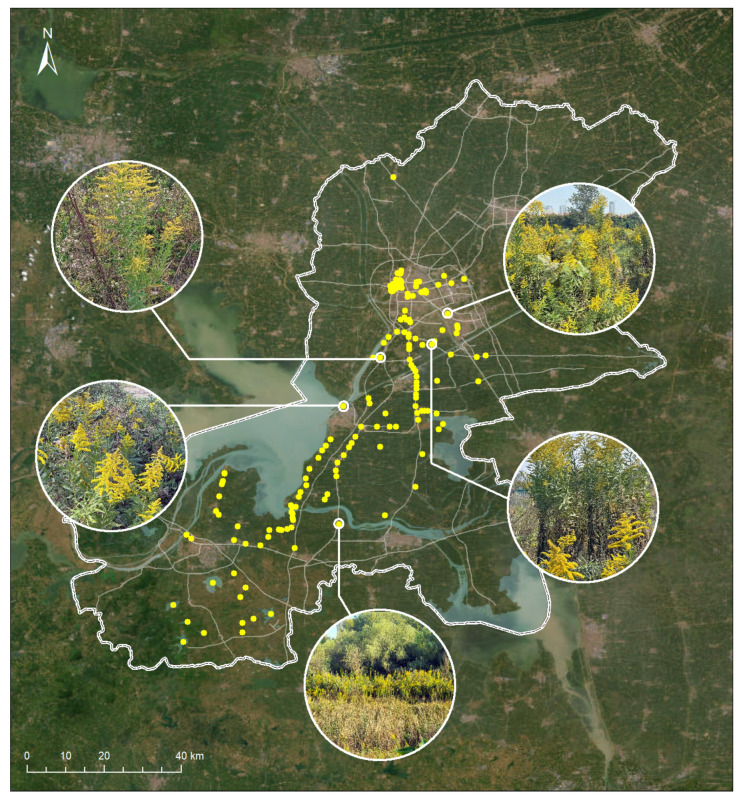
The spatial distribution of *Solidago canadensis* in Huai’an, China, was mapped using data from the EyeInvaS system.

A total of 1683 valid submissions were collected. All top-1 predictions had confidence levels exceeding 80% and were confirmed as accurate by expert reviewers. Based on geotagged records, we mapped the spatial distribution of *S. canadensis* (Figure 6), which revealed a concentration along riverbanks and transportation corridors—areas commonly associated with anthropogenic disturbance and propagule pressure.

This case study demonstrates the system’s effectiveness in enabling community-scale monitoring and provides empirical support for its practical deployment in urban and peri-urban ecosystems.

## 4. Discussion

The development of the EyeInvaS system demonstrates the potential of integrating deep learning and public participation to address the global challenge of invasive species monitoring. By combining a high-performance image recognition model with a user-friendly mobile interface, this study bridges the gap between technological innovation and citizen engagement. To further inform future applications and research, several key issues merit discussion.

### 4.1. Dataset Expansion and Model Generalization

Although our dataset included 54 invasive species across 6 taxonomic groups and incorporated synthetic augmentation to increase diversity, the current coverage remains limited. Microbial taxa were excluded, and image samples for amphibians and reptiles were relatively scarce, which may constrain model generalizability. Notably, factors such as background lighting, shadows and edge blending, which were not simulated in the synthetic data of this study, may also affect recognition efficiency, and future work will optimize these factors to enhance model generalization.

Future efforts should aim to expand taxonomic coverage, particularly for less observable groups, by integrating environmental metadata (e.g., habitat type, seasonality) alongside image data to enhance contextual inference. In addition, semi-supervised approaches such as pseudo-labeling or self-training can leverage unlabeled user-submitted data to address class imbalance and improve recognition of rare or long-tailed species 2023 [31,32].

### 4.2. Spatial Scaling via UAV Integration

Currently, EyeInvaS relies primarily on user-driven point data, which limits its coverage at regional scales. Particularly in scenarios such as lakes and marshes with complex terrain and limited accessibility, users struggle to conduct close-range observation and recording, directly resulting in sampling gaps in monitoring data.

A promising direction involves integrating public ground observations with UAV-based aerial monitoring [33]. This multi-source framework leverages the complementary strengths of crowd-sourced data and drone-enabled sensing to achieve scalable, high-resolution surveillance. Succeeding with this approach involves standardizing UAV-collected imaging and spectral data formats, aligning them with the system’s geotagging structure for spatiotemporal consistency, using the lightweight YOLOv8 detector to locate suspected IAS patches, then applying the core EfficientNetV2 model for fine-grained classification to balance speed and accuracy, and enabling real-time on-site data processing via embedded edge computing, with only high-value information transmitted back to reduce costs and delays. This integration enables rapid field deployment, expands IAS surveillance coverage, and enhances the EyeInvaS system’s performance in regional ecological monitoring [34,35].

### 4.3. Policy Interfaces and Institutional Integration

The long-term effectiveness of citizen science depends on its integration into formal ecological governance frameworks. The case study in Huai’an illustrates how citizen-contributed data can reveal spatial correlations between invasive spread and anthropogenic corridors, providing micro-level evidence to support policy intervention.

We recommend linking EyeInvaS with national and local IAS databases through standardized data protocols and review mechanisms. Among these, the standardized protocols will unify core data fields by aligning IAS taxonomic labels with official nomenclature, setting valid thresholds for AI recognition confidence, and ensuring geographic coordinates meet official spatial precision standards; the review mechanism can adopt a two-stage model, where AI first conducts preliminary screening to filter out invalid data such as submissions with missing location information or blurred images, and experts then verify data accuracy—with extra attention to species that share similar morphological features.

Inspired by global platforms such as iNaturalist and EDDMapS, incentive systems including contributor badges or leaderboards could improve user retention and data submission consistency. Such mechanisms, through clear forms of recognition, allow users to perceive the practical value of their participation in ecological monitoring, thereby enabling them to more proactively maintain their participation frequency; specific forms may include contributor badges for first observations or rare species discoveries, as well as participation leaderboards that showcase regional contribution levels.

## 5. Conclusions

This study introduces EyeInvaS, a deep learning-powered intelligent recognition system that enables convenient identification of invasive species. By leveraging neural networks and mobile technologies, we enhance the ability of non-specialist users to accurately identify invasive species.

We constructed a novel image dataset covering 54 invasive species of management priority in China and systematically evaluated nine mainstream deep learning models. EfficientNetV2 was identified as the optimal backbone. Through controlled experiments on object scale and background complexity, we revealed key factors affecting model performance and informed practical image acquisition strategies. These findings were embedded into the app’s framing guide for improved user input. The EyeInvaS system integrates image acquisition, species recognition, geotagging, and data sharing in a closed-loop workflow and demonstrated real-world efficacy in a field case study in Huai’an, China.

Future work will focus on dataset expansion and cross-platform integration, as well as institutional adoption pathways. This study contributes a scalable, replicable framework for real-time, public-powered surveillance of invasive species and offers a practical tool for biodiversity conservation and biosecurity.

## Figures and Tables

**Table 1:** Cross-taxonomic performance metrics of EfficientNetV2.

Taxonomic Group	Accuracy	Precision	Recall	F1-Score
plants	0.93	0.97	0.92	0.95
insects	0.93	0.99	0.92	0.95
mollusks	0.95	1	0.95	0.97
fishes	0.98	1	0.97	0.98
amphibians	1	1	1	1
reptiles	0.97	1	0.96	0.96

## Data Availability

The raw data supporting the conclusions of this article will be made available by the authors on request.

## Supplementary Materials

The following supporting information can be downloaded at https://zenodo.org/records/17170016, Video S1: A brief introduction of the EyeInvaS System.

## Appendix A

**Table A1:** Information and quantitative data of the database images.

Class	Family	Genus	Species Common Name	Species Scientific Name	Number of Images
C-1	Asteraceae	Ageratina	Crofton weed	*Ageratina adenophora*	48
C-2	Asteraceae	Ageratum	Billygoat weed	*Ageratum conyzoides*	205
C-3	Amaranthaceae	Alternanthera	alligator weed	*Alternanthera philoxeroides*	141
C-4	Amaranthus palmeri	Amaranthus	Dioecious amaranth	*Amaranthus palmeri*	25
C-5	Amaranthaceae	Amaranthus	Spiny amaranth	*Amaranthus spinosus*	75
C-6	Asteraceae	Ambrosia	Common ragweed	*Ambrosia artemisiifolia*	53
C-7	Asteraceae	Ambrosia	Great ragweed	*Ambrosia trifida*	48
C-8	Basellaceae	Anredera	Madeira vine	*Anredera cordifolia*	121
C-9	Poaceae	Avena	Common wild oat	*Avena fatua*	53
C-10	Asteraceae	Bidens	Hitch hikers	*Bidens pilosa*	142
C-11	Cabombaceae	Cabomba	Carolina fanwort	*Cabomba caroliniana*	63
C-12	Poaceae	Cenchrus	Spiny burr grass	*Cenchrus longispinus*	62
C-13	Asteraceae	Chromolaena	Siam weed	*Chromolaena odorata*	75
C-14	Pontederiaceae	Pontederia	Common water hyacinth	*Pontederia crassipes*	266
C-15	Asteraceae	Erigeron	Canadian horseweed	*Erigeron canadensis*	39
C-16	Asteraceae	Erigeron	White horseweed	*Erigeron sumatrensis*	36
C-17	Asteraceae	Flaveria	Coastal plain yellowtops	*Flaveria bidentis*	121
C-18	Convolvulaceae	Ipomoea	Messina creeper	*Ipomoea cairica*	283
C-19	Asteraceae	Cyclachaena	Giant sumpweed	*Cyclachaena xanthiifolia*	19
C-20	Verbenaceae	Lantana	Wild sage	*Lantana camara*	592
C-21	Asteraceae	Lactuca	Prickly lettuce	*Lactuca serriola*	53
C-22	Asteraceae	Mikania	Bitter vine	*Mikania micrantha*	39
C-23	Fabaceae	Mimosa	Flowering tree	*Mimosa bimucronata*	97
C-24	Asteraceae	Parthenium	Whitetop weed	*Parthenium hysterophorus*	14
C-25	Phytolaccaceae	Phytolacca	American pokeweed	*Phytolacca americana*	504
C-26	Araceae	Pistia	Water cabbage	*Pistia stratiotes*	67
C-27	Asteraceae	Praxelis	False boneset	*Praxelis clematidea*	85
C-28	Cucurbitaceae	Sicyos	Star-cucumber	*Sicyos angulatus*	24
C-29	Solanaceae	Solanum	Buffalobur nightshade	*Solanum rostratum*	40
C-30	Asteraceae	Solidago	Canada goldenrod	*Solidago canadensis*	527
C-31	Poaceae	Sorghum	Johnson grass	*Sorghum halepense*	14
C-32	Poaceae	Sporobolus	Smooth cordgrass	*Sporobolus alterniflorus*	73
C-33	Asteraceae	Xanthium	Spiny cocklebur	*Xanthium spinosum*	28
C-34	Tortricidae	Cydia	Apple worm	*Cydia pomonella*	26
C-35	Curculionidae	Dendroctonus	Red turpentine beetle	*Dendroctonus valens*	22
C-36	Erebidae	Hyphantria	Fall webworm	*Hyphantria cunea*	78
C-37	Chrysomelidae	Leptinotarsa	Colorado potato beetle	*Leptinotarsa decemlineata*	58
C-38	Agromyzidae	Liriomyza	Vegetable leaf miner	*Liriomyza sativae*	21
C-39	Brachyceridae	Lissorhoptus	Rice water weevil	*Lissorhoptrus oryzophilus*	30
C-40	Matsucoccidae	Matsucoccus	Japanese pine bast scale	*Matsucoccus matsumurae*	9
C-41	Pseudococcidae	Oracella	Loblolly pine mealybug	*Oracella acuta*	105
C-42	Pseudococcidae	Phenacoccus	Cotton mealybug	*Phenacoccus solenopsis*	47
C-43	Curculionidae	Rhynchophorus	Red palm weevil	*Rhynchophorus ferrugineus*	47
C-44	Formicidae	Solenopsis	Red imported fire ant	*Solenopsis invicta*	111
C-45	Noctuidae	Spodoptera	Fall armyworm	*Spodoptera frugiperda*	254
C-46	Gelechiidae	Tuta	South american tomato pinworm	*Tuta absoluta*	27
C-47	Achatinidae	Lissachatina	Giant African land snail	*Lissachatina fulica*	138
C-48	Ampullariidae	Pomacea	Golden apple snail	*Pomacea canaliculata*	166
C-49	Lepisosteidae	Atractosteus	Alligator gar	*Atractosteus spatula*	317
C-50	Loricariidae	Pterygoplichthys	Amazon sailfin catfish	*Pterygoplichthys pardalis*	77
C-51	Cichlidae	Coptodon	Redbelly tilapia	*Coptodon zillii*	44
C-52	Ranidae	Lithobates	American bullfrog	*Lithobates catesbeianus*	144
C-53	Chelydridae	Macrochelys	Alligator snapping turtle	*Macrochelys temminckii*	106
C-54	Emydidae	Trachemys	Red-eared slider	*Trachemys scripta elegans*	250

**Table A2:** Attention localization analysis for misclassified images with Grad-CAM and guided Grad-CAM.

Input Image	Grad-CAM	Guided Grad-CAM	Input Image	Grad-CAM	Guided Grad-CAM
					
					
					
					

## References

[1] Hulme P.E. (2009). Trade, transport and trouble: Managing invasive species pathways in an era of globalization. J. Appl. Ecol..

[2] Pyšek P., Hulme P.E., Simberloff D., Bacher S., Blackburn T.M., Carlton J.T., Dawson W., Essl F., Foxcroft L.C., Genovesi P. (2020). Scientists’ warning on invasive alien species. Biol. Rev..

[3] IPBES (2023). Thematic Assessment Report on Invasive Alien Species and Their Control.

[4] Venette R.C., Hutchison W.D. (2021). Invasive Insect Species: Global Challenges, Strategies & Opportunities. Front. Insect Sci..

[5] Bellard C., Cassey P., Blackburn T.M. (2016). Alien species as a driver of recent extinctions. Biol. Lett..

[6] Valiente-Banuet A., Aizen M.A., Alcántara J.M., Arroyo J., Cocucci A., Galetti M., García M.B., García D., Gómez J.M., Jordano P. (2015). Beyond species loss: The extinction of ecological interactions in a changing world. Funct. Ecol..

[7] Diagne C., Leroy B., Vaissière A.-C., Gozlan R.E., Roiz D., Jarić I., Salles J.-M., Bradshaw C.J., Courchamp F. (2021). High and rising economic costs of biological invasions worldwide. Nature.

[8] Turbelin A.J., Cuthbert R.N., Essl F., Haubrock P.J., Ricciardi A., Courchamp F. (2023). Biological invasions are as costly as natural hazards. Perspect. Ecol. Conser..

[9] Vilà M., Dunn A.M., Essl F., GÓmez-DÍaz E., Hulme P.E., Jeschke J.M., NÚÑez M.A., Ostfeld R.S., Pauchard A., Ricciardi A. (2021). Viewing emerging human infectious epidemics through the lens of invasion biology. Bioscience.

[10] Rishan S.T., Kline R.J., Rahman M.S. (2023). Applications of environmental DNA (eDNA) to detect subterranean and aquatic invasive species: A critical review on the challenges and limitations of eDNA metabarcoding. Environ. Adv..

[11] Thomas A.C., Tank S., Nguyen P.L., Ponce J., Sinnesael M., Goldberg C.S. (2020). A system for rapid eDNA detection of aquatic invasive species. Environ. DNA.

[12] Lopatin J., Dolos K., Kattenborn T., Fassnacht F.E. (2019). How canopy shadow affects invasive plant species classification in high spatial resolution remote sensing. Remote Sens. Ecol. Conserv..

[13] Clavijo McCormick A., Effah E., Najar-Rodriguez A. (2023). Ecological aspects of volatile organic compounds emitted by exotic invasive plants. Front. Ecol. Evol..

[14] Crall A.W., Jordan R., Holfelder K., Newman G.J., Graham J., Waller D.M. (2012). The impacts of an invasive species citizen science training program on participant attitudes, behavior, and science literacy. Public Underst. Sci..

[15] Aristeidou M., Herodotou C., Ballard H.L., Young A.N., Miller A.E., Higgins L., Johnson R.F. (2021). Exploring the participation of young citizen scientists in scientific research: The case of iNaturalist. PLoS ONE.

[16] Wallace R.D., Bargeron C.T., Ziska L.H. (2022). Identifying Invasive Species in Real Time: Early Detection and Distribution Mapping System (EDDMapS) and Other Mapping Tools. Invasive Species and Global Climate Change.

[17] Krizhevsky A., Sutskever I., Hinton G.E. ImageNet classification with deep convolutional neural networks. Proceedings of the 25th International Conference on Neural Information Processing Systems.

[18] He K., Zhang X., Ren S., Sun J. Deep residual learning for image recognition. Proceedings of the 2016 IEEE Conference on Computer Vision and Pattern Recognition (CVPR).

[19] Dosovitskiy A., Beyer L., Kolesnikov A., Weissenborn D., Zhai X., Unterthiner T., Dehghani M., Minderer M., Heigold G., Gelly S. (2020). An image is worth 16x16 words: Transformers for image recognition at scale. arXiv.

[20] Liu Z., Lin Y., Cao Y., Hu H., Wei Y., Zhang Z., Lin S., Guo B. Swin transformer: Hierarchical vision transformer using shifted windows. Proceedings of the 2021 IEEE/CVF International Conference on Computer Vision (ICCV).

[21] Zhang X., Zhou X., Lin M., Sun J. Shufflenet: An extremely efficient convolutional neural network for mobile devices. Proceedings of the 2018 IEEE/CVF Conference on Computer Vision and Pattern Recognition (CVPR).

[22] Tan M., Le Q.V. Efficientnet: Rethinking model scaling for convolutional neural networks. Proceedings of the 36th International Conference on Machine Learning (ICML).

[23] Howard A.G., Zhu M., Chen B., Kalenichenko D., Wang W., Weyand T., Andreetto M., Adam H. (2017). MobileNets: Efficient Convolutional Neural Networks for Mobile Vision Applications. arXiv.

[24] Binta Islam S., Valles D., Hibbitts T.J., Ryberg W.A., Walkup D.K., Forstner M.R.J. (2023). Animal Species Recognition with Deep Convolutional Neural Networks from Ecological Camera Trap Images. Animals.

[25] Goodwin M., Halvorsen K.T., Jiao L., Knausgård K.M., Martin A.H., Moyano M., Oomen R.A., Rasmussen J.H., Sørdalen T.K., Thorbjørnsen S.H. (2022). Unlocking the potential of deep learning for marine ecology: Overview, applications, and outlook. ICES J. Mar. Sci..

[26] Lopez-Vazquez V., Lopez-Guede J.M., Chatzievangelou D., Aguzzi J. (2023). Deep learning based deep-sea automatic image enhancement and animal species classification. J. Big Data.

[27] Sahu K., Minz S. (2023). Adaptive Segmentation with Intelligent ResNet and LSTM–DNN for Plant Leaf Multi-disease Classification Model. Sens. Imaging.

[28] Reddy S.R.G., Varma G.P.S., Davuluri R.L. (2024). Deep Neural Network (DNN) Mechanism for Identification of Diseased and Healthy Plant Leaf Images Using Computer Vision. Ann. Data. Sci..

[29] Simonyan K., Zisserman A. (2014). Very deep convolutional networks for large-scale image recognition. arXiv.

[30] Huang G., Liu Z., Van Der Maaten L., Weinberger K.Q. Densely connected convolutional networks. Proceedings of the 2017 IEEE Conference on Computer Vision and Pattern Recognition (CVPR).

[31] Ferreira R.E.P., Lee Y.J., Dórea J.R.R. (2023). Using pseudo-labeling to improve performance of deep neural networks for animal identification. Sci. Rep..

[32] Jia Y., Li S.S., Guo X., Lei B., Hu J.Q., Xu X.H., Zhang W. (2022). Selfee, self-supervised features extraction of animal behaviors. eLife.

[33] Gonçalves C., Santana P., Brandão T., Guedes M. (2022). Automatic detection of Acacia longifolia invasive species based on UAV-acquired aerial imagery. Inf. Process. Agric..

[34] Cruz C., McGuinness K., Perrin P.M., O’Connell J., Martin J.R., Connolly J. (2023). Improving the mapping of coastal invasive species using UAV imagery and deep learning. Int. J. Remote Sens..

[35] Bergamo T.F., de Lima R.S., Kull T., Ward R.D., Sepp K., Villoslada M. (2023). From UAV to PlanetScope: Upscaling fractional cover of an invasive species Rosa rugosa. J. Environ. Manag..

